# Extraction and characterization of microcrystalline cellulose from kelp (*Laminaria japonica*) waste

**DOI:** 10.1371/journal.pone.0331699

**Published:** 2026-06-30

**Authors:** Zhenhua Liu, Wei Xie, Lan Ye, Chaojie Chen

**Affiliations:** 1 School of Environmental and Food Engineering, Liuzhou Polytechnic University, Liuzhou, Guangxi, China; 2 School of Food and Pharmaceutical Engineering, Wuzhou University, Wuzhou, Guangxi, China; Universidad Tecnica de Ambato, ECUADOR

## Abstract

Kelp, an economically important brown alga widely cultivated in East Asia, produces kelp waste rich in cellulose as a byproduct of alginate extraction. In this study, kelp cellulose (KC) was first extracted from the kelp waste. Subsequently, 2 wt% hydrochloric acid was used to hydrolyze the kelp cellulose at 100 °C for 30 min to isolate kelp microcrystalline cellulose (KMCC) from the KC. Characterization by X-ray diffraction (XRD) and Fourier transform infrared spectroscopy (FTIR) revealed that the chemical structures and cellulose I crystal form remained intact during hydrolysis, with the crystallinity index increasing from 76.4% to 78.1%. Thermogravimetric analysis (TGA) and derivative TGA indicated enhanced thermostability of KMCC compared to KC. Scanning electron microscopy (SEM) observations showed that acid hydrolysis transformed the irregular thin-sheet structure of KC into smaller-sized KMCC with a morphology distinct from the rod-like structure of commercial microcrystalline cellulose (CMCC). The level-off degree of polymerization (LODP) of KMCC measured around 164.3, closely resembling the value for CMCC at 166.5, with a yield of 85.5%. This study proposes a novel method to utilize kelp waste by converting it into MCC, offering a potential avenue for its valorization.

## Introduction

Cellulose, the widespread renewable natural polymer in nature, is biosynthesized by a large number of organisms such as plants, algae and bacteria on the Earth. Cellulose has fulfilled the needs of mankind since ancient times, serving purposes ranging from providing warmth through burning to becoming a raw material for various modern applications such as papermaking, food production, clothing, and additives. Cellulose chains are biosynthesized by linking β-D-glucose units through 1–4 glycosidic bonds in nature. These cellulose molecules then self-organize into microfibrils, which cluster together to form larger cellulose fibers. Cellulose can be processed into various derivatives. Via acid hydrolyzing, two major crystals obtained maintaining the crystallinity are classified as microcrystalline cellulose (MCC) and nanocrystalline cellulose (NCC) based on their dimensional differences. This study focused on the MCC, a purified and partially depolymerized cellulose.

The size of cellulose molecules is commonly indicated by the degree of polymerization (DP), which denotes the number of glucose residues in each cellulose chain. Native cellulose molecules have a broad DP range, reaching up to 15,000 in cotton fiber, whereas the typical DP for MCC is less than 350 [1]. Cellulose microfibrils consist of both crystalline and amorphous regions. In the amorphous regions, the cellulose molecules are less densely packed, making them more susceptible to external factors and chemical modifications. Various methods, including chemical, physical, biological, and combined processes, can be employed to selectively degrade the amorphous regions and produce MCC [2]. Among these, the most extensively studied and industrially established method involves hydrolyzing cellulose with mineral acids, followed by neutralization and drying of the resulting aqueous slurry. Numerous researchers have produced MCC from various cellulose sources and studied the impact of processing conditions on its physical and chemical properties. Leppänen et al. [3] isolated MCC from cotton, flax and woods and found the obtained MCC had similar supermolecular structure. El-Sakhawy et al. [4] isolated MCC from agricultural residues and demonstrated that the particle size was affected by the nature of acid. De Oliveira et al. [5] prepared MCC from bacterial cellulose and indicated that the MCC had an average particle size of 70–90 μm and DP of 250, while crystalline structure changed. Trache et al. [6] harvested MCC from *esparto* grass fibres and described that the MCC was more crystalline than native cellulose, with a DP of 318. Given its distinct physical and chemical properties, MCC finds a wide range of applications, including in polymer composites, food, pharmaceuticals, cosmetics, and construction materials [7].

Kelp (*Laminaria japonica*) is a large seaweed primarily cultivated in China, Korea, and Japan, with China contributing over 80% of the global annual production. Taxonomically, kelp belongs to the brown algae (Phaeophyceae). The divergence between aquatic algae and vascular plants occurred over a billion years ago, after which kelp evolved unique components such as alginate, mannitol, and iodine, though there is no conclusive evidence of lignin presence [8]. Beyond its use as a vegetable, kelp plays a crucial role as a source of alginate. In China, numerous mills extract alginate from kelp, producing sodium alginate as a final product. The byproduct of alginate extraction, known as kelp waste, is rich in minerals, protein, and cellulose, making it valuable for use in animal feed and mariculture. Zheng et al. [9] discovered that kelp waste extracts could enhance the biomass productivity of certain microalgae, particularly Chlorella strains. Later, Zheng et al. [10] suggested that adding an appropriate amount of acetate to kelp waste extracts could be an effective method for cultivating *C. sorokiniana* for biofuel production.

Cellulose is abundant in kelp waste [11], yet its potential for high value applications remains underexplored. While MCC is widely produced from terrestrial biomass, research on algae derived MCC remains limited. Exploring MCC from kelp waste could provide new insights into its properties and expand its applications. This study investigates the preparation and characteristics of MCC from kelp waste, highlighting its potential as an alternative MCC source and contributing to the efficient utilization of marine biomass resources.

## Materials and methods

### Materials

Kelp (*Laminaria japonica*) was purchased from Yujia Seafood Products Co., Ltd. in Xiapu, Fujian, China. Commercial MCC was obtained from Qufu Tianli Medical Supplements Co., Ltd. (China). The chemicals utilized in this study were of analytical grade and were employed directly without any additional purification.

### Preparation of kelp MCC

Alginate was first extracted from the purchased kelp using Na_2_CO_3_ to obtain the kelp waste. The acid soluble ash was then removed by treating the waste with hydrochloric acid. Subsequently, the waste was treated with 3 wt% NaOH at a solid to liquid ratio of 1:10 (g/mL) at 100 °C for 140 min to obtain the kelp cellulose. The kelp cellulose was hydrolyzed using 2 wt% hydrochloric acid at 100 °C for 30 min, with a solid to liquid ratio of 1:20 (g/mL). The mixture was then filtered under suction and rinsed with deionized water until a stable pH was reached. Finally, the material was dried in a vacuum oven at 55 °C and ground to produce kelp derived MCC for further use.

### Degree of polymerization (DP) and water retention value (WRV)

The degree of polymerization was determined by intrinsic viscosity measurements according to the Chinese Pharmacopoeia 2015 for microcrystalline cellulose, using a 1 M solution of copper (II)-ethylenediamine complex in water, purchased from Acros Organics, UK.

Water retention value was measured following the method described by Cheng et al. [12]. A specific amount of water saturated pulp was placed in a separator tube with a 200 mesh screen and centrifuged for 15 min at 2000 rpm. Afterward, the pulp was taken out and measured mass (*m*_*1*_), and then was placed in an oven (at 105 °C) to dry until a constant weight (*m*_*0*_). WRV was calculated based on the following equation:


$$WRV=\frac{m_{1}-m_{0}}{m_{0}}\times 100\%$$ (1)

### Statistical analysis

Each variation point for the DP, WRV, and MCC yield tests was repeated three times. The results are expressed as mean values ± SD.

### Characterization

#### Fourier transform infrared spectroscopy (FTIR).

FTIR spectrum (4000−400 cm^−1^) of the sample was recorded using a Nicolet iS5 spectrometer with an iD7 attenuated total reflectance (ATR) accessory.

#### X-ray diffraction (XRD).

XRD patterns were obtained using an X-ray powder diffractometer (X’Pert Pro, Netherlands) with Cu Kα radiation (λ = 1.5418 Å), operating at 40 kV and 40 mA. The scanning range was set from 10° to 60° at a rate of 5°/min.

The crystallinity index (CI) was determined using the empirical equation [13].


$$CI(\%)=\frac{I_{002}-I_{am}}{I_{002}}$$ (2)

Here *I*_*002*_ represents the maximum diffraction intensity of crystalline from plane (002) at 2θ = 22.6° and *I*_*am*_ is the background scatter intensity measured at 2θ = 18°.

#### Thermogravimetric analysis (TGA).

TGA was conducted on the specimens in a nitrogen gas atmosphere (flow rate: 50 mL/min) over the temperature range of 35–900 °C, with a heating rate of 10 °C/min by a thermogravimetric analyzer (Mettler Toledo TGA/DSC1, microbalance precision ± 0.01 μg). The typical sample weight was 5 mg.

#### Particle size distribution.

The particle size properties were assessed using a Malvern Instruments Mastersizer 2000 laser diffraction particle size analyzer. Prior to the size measurement, an aqueous particle suspension underwent a 5 minute ultrasound treatment at 80 W and a frequency of 50 kHz.

#### Morphology.

SEM observation was performed using a ZEISS EVO 18 scanning electronic microscope at an acceleration voltage of 3 kV and a typical WD of 8.5 mm. The samples were coated with gold before observation.

## Results and discussion

### Characterization

#### X-ray diffraction.

**Fig 1:**
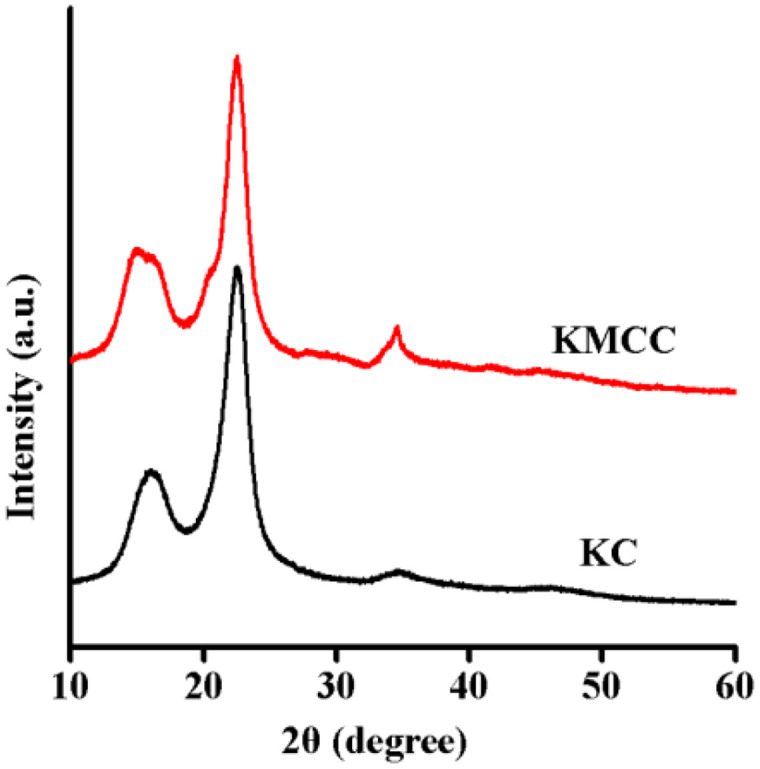
XRD patterns of kelp cellulose (KC) and kelp MCC (KMCC).

X-ray diffraction (XRD) was employed to characterize the crystal form and crystallinity index of the cellulose and its derivative crystals. The XRD patterns of KC and KMCC, as shown in Fig 1, exhibit characteristic peaks at 2θ = 14.7°, 16.4°, 22.7°, and 34.6°, corresponding to the ($1\overset{―}{1}0$), (110), (200), and (004) planes of the typical cellulose I structure [14]. This indicates that the crystal form was preserved during the acid hydrolysis process used to prepare KMCC. In contrast, some studies have reported the formation of cellulose II in cellulose derivatives prepared through acid hydrolysis, such as MCC derived from oil palm empty fruit bunch fiber [15], cellulose nanowhiskers isolated from oil palm biomass MCC [16], and nanocellulose obtained from waste sugarcane bagasse [17].

The crystallinity index figured out based on empirical equation and XRD data are 76.4% and 78.1% for KC and KMCC, respectively. The increase of crystallinity index from KC to KMCC is ascribed to the hydrolysis of the amorphous regions of KC, which is confirmed by the WRV results of the crystals in Table 1. The WRV for KMCC decreased notably to 133.70% from 407.5% for KC, whereas it was only slightly different from the value for CMCC (119.9%). In cellulose, the amorphous regions contain more exposed hydroxyl groups than the crystalline regions, leading to a higher affinity for water molecules and, consequently, an elevated WRV. During acid hydrolysis, these amorphous regions are more susceptible to degradation, resulting in a decrease in WRV. This degradation also contributes to an increase in the crystallinity index, as the proportion of crystalline regions becomes more prominent. The crystallinity index of KMCC isolated in our study was higher than that of MCC (77.6%) derived from tamarind (*Tamarindus indica*) seeds [18] and MCC (66.4%) obtained from *Ficus benghalensis* leaves [19]. This difference can be attributed to variations in the cellulose source and the specific isolation and purification methods used.

**Table 1:** WRV and DP of kelp cellulose, kelp MCC and commercial MCC.

	Water retention value (%)	Degree of polymerization
**Kelp cellulose**	407.5 ± 8.0	936.0 ± 30.0
**Kelp MCC**	133.7 ± 4.6	164.3 ± 2.1
**Commercial MCC**	119.9 ± 4.2	166.5 ± 1.7

#### FTIR analysis.

**Fig 2:**
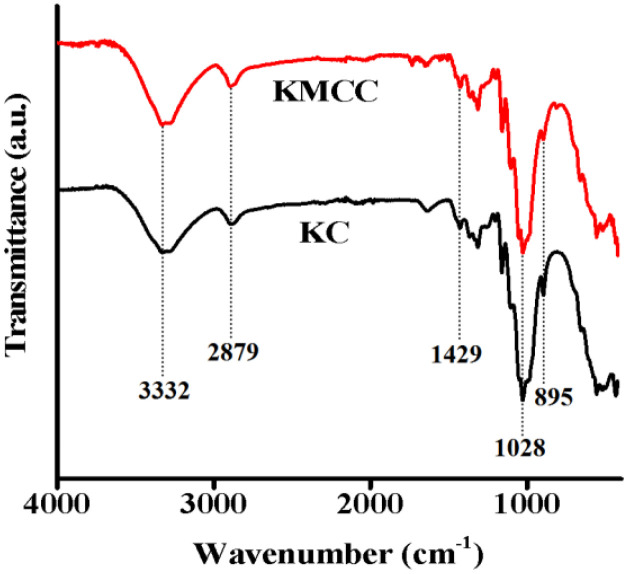
FTIR spectra of kelp cellulose (KC) and kelp MCC (KMCC).

It is widely recognized that Fourier transform infrared spectroscopy (FTIR) is often used to determine the chemical structures of samples. The FTIR spectra of KC and KMCC are shown in Fig 2. In both spectra, the broad absorption bands observed between 3600 and 3400 cm ⁻ ¹ correspond to the stretching vibrations of -OH groups [20], while the absorption at 2879 cm ⁻ ¹ is attributed to C-H stretching vibrations [21]. The additional absorption peaks observed at 1429 cm ⁻ ¹, 1028 cm ⁻ ¹, and 895 cm ⁻ ¹ are primarily associated with intermolecular hydrogen bonding at the C6 group, C-O-C pyranose ring skeletal vibrations, and C-H rocking vibrations, respectively. The characteristic absorptions at 3332, 1429, 1028, and 895 cm ⁻ ¹ in both spectra are indicative of cellulose [16].

During hydrolysis, acid was employed to target the breakage of glucosidic bonds in cellulose under controlled acid concentration. Typically, the chemical structures of cellulose remained unchanged in the process, whether isolating MCC or NCC from its cellulose precursor. This has been confirmed by numerous previous literature reports, such as the production of MCC from oil palm biomass [13,15,16], the isolation of MCC from various agricultural residues [4], and the preparation of NCC from sisal fibers [22]. The FTIR analysis confirms that the acid concentration used in our study was effective for isolating MCC from kelp cellulose without altering the chemical structures of the crystals.

#### Thermogravimetric analysis.

**Fig 3:**
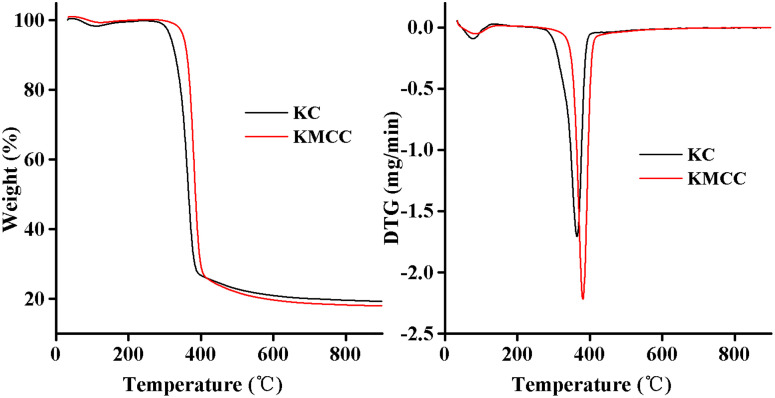
TGA and DTG curves of kelp cellulose (KC) and kelp MCC (KMCC).

Thermogravimetric analysis (TGA) and derivative thermogravimetric analysis (DTG) were used to characterize the thermal decomposition behavior of KC and KMCC, and the results are graphically presented in Fig 3. According to the DTG curves, both KC and KMCC gave a thermal event around 60 °C, attributed to the evaporation of moisture bound to the surface of the specimens [23,24]. The other major peaks in the two DTG curves, covering the range from 220 to 400 °C, are associated with the decomposition of cellulose component, specifically cellulose chain scissions resulting from the cleavage of C-C and C-O bonds [25]. Furthermore, the major peak for KMCC reaches its maximum at 381 °C, which is higher than that of KC (365 °C). This is attributed to the former’s higher crystallinity resulting from the removal of amorphous regions by hydrolysis, making it more resistant to thermal degradation. MCCs with higher crystallinity exhibited greater thermal stability. Similar phenomena were also observed when producing MCCs from pomelo peel [26], corn stover [27] and date seeds [28].

Compared to cellulose and crystals derived from raw materials with low ash content, those obtained from the high ash content kelp waste exhibited a relatively high residual mass (Fig 3, TGA). A similar trend is also evident in the case of rice husks, where both cellulose and crystals displayed a high residual mass (around 20%) [29,30]. Additionally, KMCC showed a lower residual mass in TGA analysis compared to KC, reflecting the higher purity of cellulose in MCC [31]. The high residual weight of KC is primarily attributed to the presence of flame retardant compounds, which promote char formation [32,33].

#### Morphology.

**Fig 4:**
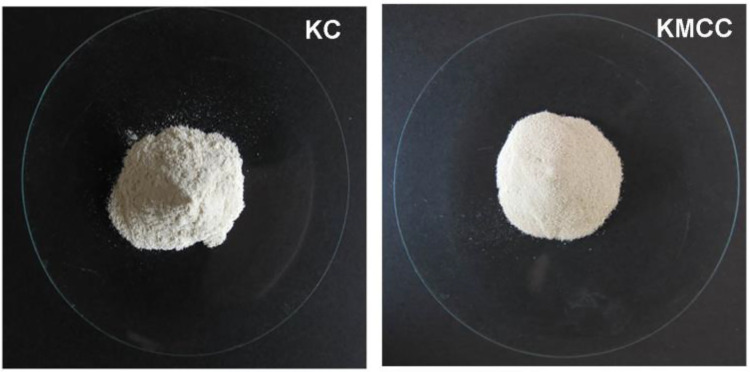
Photographs of kelp cellulose (KC) and kelp MCC (KMCC).

**Fig 5:**
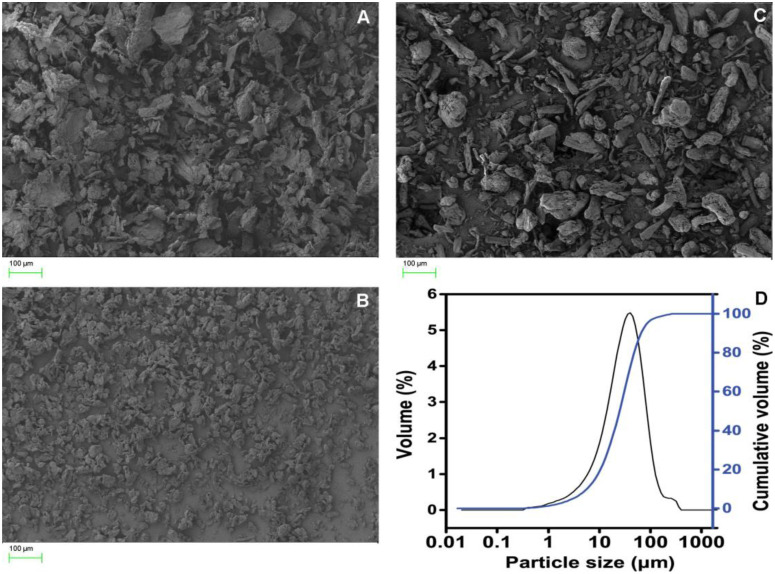
SEM images and particle size distribution of kelp microcrystalline cellulose. A, B and C are SEM images of kelp cellulose, kelp MCC and commercial MCC, respectively. D is particle size distribution of kelp MCC.

To the naked eye, the prepared KMCC appeared nearly identical to the original kelp cellulose (KC); however, KC seemed fluffier in comparison (Fig 4). This difference in texture could be attributed to the higher crystallinity of KMCC, as confirmed by XRD results, which contributed to its more granular appearance. The morphologies of KC and KMCC were thoroughly examined using SEM and compared to CMCC. Both KC and KMCC exhibited irregular thin-sheet structures with rough surfaces (Fig 5A and 5B). However, there are significant differences between KMCC and its precursor KC, particularly in terms of particle size. During hydrolysis, the acid cleaved the glycosidic bonds in KC, leading to its fragmentation into smaller particles. This reduction in particle size resulted in KMCC being smaller than its precursor, KC. Moreover, compared to KC, KMCC displayed better monodispersity. Further analysis of the particle size distribution of KMCC revealed a coverage range from 1 to 300 μm, with a d_50_ value of 28 μm (Fig 5D).

Characteristics of MCC such as shape and particle size are different based on both their hydrolysis conditions and cellulose sources [4]. In our studies, the morphologies of KMCC and CMCC from pinewood exhibited significant differences. KMCC exhibited an irregular thin-sheet structure, while CMCC predominantly showcased a rod-like shape with larger dimensions (Fig 5C). This structural contrast can be linked to the distinctive cellular and chemical composition of kelp, characterized by the absence of robust cellulose fibers and cement lignins. Furthermore, this compositional distinction contributes significantly to the soft body of kelp, deviating from the more common upright and sturdy growth patterns typically observed in terrestrial plants.

### Effect of preparation variables on DP and yield of kelp MCC

The degree of polymerization (DP) of the extracted kelp cellulose was 936.0, markedly surpassing the commercial MCC, which had a DP of 166.5 (Table 1). Hydrolysis is essential for transferring the KC into KMCC. Sulphuric acid and hydrochloric acid are the two most commonly used acids in the preparation of MCC [2]. Hydrochloric acid is milder and more cost effective, therefore used in industrial production and our study to hydrolyze the kelp cellulose. HCl concentration and reaction time are dominant factors in kelp cellulose hydrolysis and were therefore thoroughly investigated.

**Fig 6:**
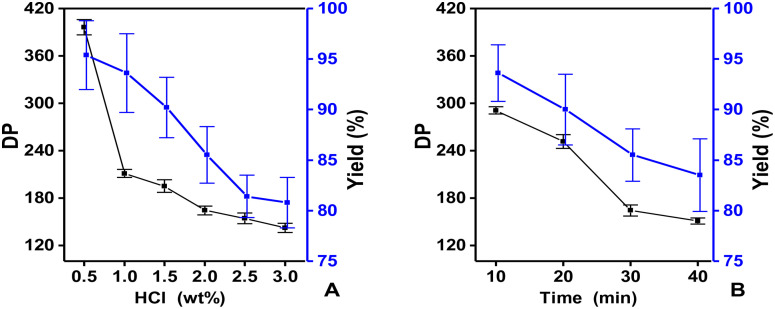
Effect of hydrochloric acid concentration and hydrolysis time on DP and yield of kelp MCC.

Hydrolysis rate is known to decrease as the DP reaches a specific level referred to as the “level-off degree of polymerization” (LODP). LODP is a characteristic of specific pulps, with values typically ranging from 180 to 300 depending on the pulp source [34]. In our studies, with the increase of HCl concentration, both the yield and DP of prepared KMCC decreased. Interestingly, when HCl concentration exceeded 1%, the decrease in DP became less pronounced (Fig 6A). This phenomenon suggests that the KMCC obtained at HCl concentrations exceeding 1% may approach or even reach its LODP. Furthermore, it is noticed that when the HCl concentration exceeded 2%, the color of the pulp darkened, possibly due to the oxidative effect of HCl. Therefore, a 2% HCl concentration was selected for the hydrolysis process to prepare KMCC. The acid concentration used to isolate KMCC was lower than that employed for the isolation of MCC from other organisms, such as 7.5% for oil palm empty fruit bunch pulp [16] or 6% for bagasse, cotton stalk and rice straw pulp [4]. The possible reason is that, unlike the typical cellulose fibers found in most terrestrial plants, kelp is primarily composed of parenchyma cells. These cells have a much smaller particle size, making them easier to hydrolyze. This is advantageous for KMCC, as it requires less chemical consumption during its preparation process.

The effect of hydrolysis time was analogous to that of HCl concentration (Fig 6B). Prolonged reaction time allows for more complete hydrolysis of the cellulose chains, thereby reducing the DP. Our experiments showed that the DP decreased rapidly with increasing reaction time up to 30 min. Extending the reaction time beyond 30 min led to marginal improvements in DP reduction, indicating it reached the LODP at 30 min. Therefore, a hydrolysis time of 30 min is optimal for achieving the desired MCC characteristics without over processing the cellulose. At this time, the DP of KMCC measured 164.3, closely resembling the value for CMCC at 166.5, with a yield of 85.5%.

## Conclusions

Kelp microcrystalline cellulose was successfully isolated by using 2 wt% hydrochloric acid to hydrolyze kelp cellulose at 100 °C for 30 min, with a solid to liquid ratio of 1:20 (g/mL). The degree of polymerization and water retention value of the isolated KMCC were comparable to those of commercial MCC. XRD characterization showed the cellulose I crystalline form was retained during the acid hydrolysis, and the crystallinity index of KMCC was 78.1%, higher than the value for kelp cellulose at 76.4%. FTIR spectra confirmed that the chemical structures remained unchanged in the KMCC preparation process. SEM observations revealed that the KMCC exhibited an irregular thin-sheet structure, whereas the predominant morphology of CMCC was rod-like in shape. It is indicated that kelp waste could potentially be used as a source material for MCC.

## Supporting information

S1 File Original data of Table 1 and Fig 6. (ZIP)
